# Top-down vs. bottom-up control on vegetation composition in a tidal marsh depends on scale

**DOI:** 10.1371/journal.pone.0169960

**Published:** 2017-02-03

**Authors:** Kelly Elschot, Anke Vermeulen, Wouter Vandenbruwaene, Jan P. Bakker, Tjeerd J. Bouma, Julia Stahl, Henk Castelijns, Stijn Temmerman

**Affiliations:** 1 Conservation Ecology Group, Groningen Institute of Evolutionary Life Sciences GELIFES, University of Groningen, CC Groningen, The Netherlands; 2 Ecosystem Management Research Group, University of Antwerp, Universiteitsplein, Wilrijk, Belgium; 3 Flanders Hydraulics Research, Flemish Government, Berchemlei, Antwerpen, Belgium; 4 NIOZ Royal Netherlands Institute for Sea Research, Department of Estuarine and Delta Systems and Utrecht University, AC Yerseke, the Netherlands; 5 Sovon, Dutch Centre for Field Ornithology, GA Nijmegen, The Netherlands; 6 *Natuurbeschermingsvereniging De Steltkluut*, AH Terneuzen, The Netherlands; Technion Israel Institute of Technology, ISRAEL

## Abstract

The relative impact of top-down control by herbivores and bottom-up control by environmental conditions on vegetation is a subject of debate in ecology. In this study, we hypothesize that top-down control by goose foraging and bottom-up control by sediment accretion on vegetation composition within an ecosystem can co-occur but operate at different spatial and temporal scales. We used a highly dynamic marsh system with a large population of the Greylag goose (*Anser anser)* to investigate the potential importance of spatial and temporal scales on these processes. At the local scale, Greylag geese grub for below-ground storage organs of the vegetation, thereby creating bare patches of a few square metres within the marsh vegetation. In our study, such activities by Greylag geese allowed them to exert top-down control by setting back vegetation succession. However, we found that the patches reverted back to the initial vegetation type within 12 years. At large spatial (i.e. several square kilometres) and temporal scales (i.e. decades), high rates of sediment accretion surpassing the rate of local sea-level rise were found to drive long-term vegetation succession and increased cover of several climax vegetation types. In summary, we conclude that the vegetation composition within this tidal marsh was primarily controlled by the bottom-up factor of sediment accretion, which operates at large spatial as well as temporal scales. Top-down control exerted by herbivores was found to be a secondary process and operated at much smaller spatial and temporal scales.

## Introduction

The relative importance of top-down control by consumers versus bottom-up control by resources on primary production within ecosystems has been the subject of a long-standing debate in ecology [1–4]. Both sides of the debate are supported by many studies, showing either the importance of top-down control by grazers or bottom-up control by environmental conditions on vegetation [5–10]. For example, herbivores are known to have potentially large impacts on vegetation [9]. They can drive vegetation composition [5,11], plant height [12,13], plant species richness [14] as well as heterogeneity within an ecosystem [15,16]. Important environmental conditions that control the vegetation bottom up are mainly nutrient availability [8,10] but also natural succession [17] or tidal regime in wetland ecosystems [18]. More recently, a growing number of studies have been showing that both factors can interact to control vegetation composition, plant diversity and/or plant standing biomass [2,19–21] in different systems, such as terrestrial habitats [19,22,23] and intertidal areas [21] as well as for primary producers in marine habitats [2,20]. Reviews conducted across ecosystems have shown that the impact of consumer versus nutrient resource control on producer biomass differ between freshwater, marine and terrestrial ecosystems [2,24]. Thus, for many ecosystems, the relative importance of each factor remains unclear. Even within the same ecosystem, the outcome can depend on the local environmental conditions of the study site [22,25,26] or the temporal [19,27] and spatial scale [28] that is considered.

Top-down control by grazing on vegetation often occurs at relatively small scales (few m^2^) by impacting the vegetation locally [9]. Bottom-up control, however, generally occurs at landscape scales (km^2^) as it is driven by large-scale factors, mainly nutrient availability [8,10]. In this study, we predict that top-down control by herbivores on the vegetation and bottom-up control through abiotic properties will co-occur but operate at different spatial and temporal scales. To test this, we studied vegetation composition and changes in ecosystem-level properties at local (a few m^2^) and landscape scales (a few km^2^) in the context of long-term ecosystem development (decades from 1979 to present). We used tidal marshes with extensive goose grazing as the study system.

Global increases in migratory goose populations has resulted in increasing grazing pressure on many tidal marshes [29–32]. Here, we focus on a large brackish tidal marsh (28 km^2^) in the southwest of the Netherlands (Saeftinghe), which provides feeding habitats for a large proportion of the Dutch population of Greylag geese, *Anser anser* [33]. These geese grub for below-ground storage organs of *Bolboschoenus maritimus* (nomenclature following Van der Meijden [34]), which are mainly present in wet depressions near the creeks. By disturbing the soil surface with grubbing, Greylag geese open up bare patches of a few m^2^ in size in the vegetation [35]. Eventually, grubbing geese can degrade large areas of marsh which may take decades to recover [36–39], although large-scale degradation has not been observed so far at our study site. Goose population size at the study site had strongly increased after 1990 due to the abandonment of hunting [40] but decreased again in the past decade, despite the absence of predators for winter-staging geese [33]. This decrease may be linked to a reduction in their main food source, *B*. *maritimus* [33].

In this study, we focus on sediment accretion as the main (bottom-up) driver even though most studies conclude that the availability of resources is the most important bottom-up factor controlling vegetation. This is because tidal marshes are highly dynamic ecosystems. Marsh vegetation slows down tidal current and thereby stimulates mineral sedimentation, resulting in accretion and a gradient of increasing surface elevation with marsh age [41–44]. If marsh elevation increases faster than sea-level rise, waterlogging of the soil decreases, which in turn facilitates vegetation succession and the increase of vegetation types bound to higher elevated or well-drained sites [45,46]. As such, marsh elevation and tidal inundation are considered the most important factors controlling vegetation composition in tidal marshes [18,41,47,48]. This dynamic character makes marshes very suitable systems to study ecosystem processes, as strong successional differences can develop in just a few decades [41].

Using the Saeftinghe tidal marsh as a model system, we propose two hypotheses: 1) top-down control by increasing numbers of grubbing Greylag geese on the vegetation results in lower cover of *Bolboschoenus maritimus* at the small temporal and local scale and 2) bottom-up control by sediment accretion on vegetation development results in higher elevation and reduction in the overall cover of *Bolboschoenus maritimus* at the large temporal and landscape scale. To address these hypotheses, we measured re-vegetation of depressions grubbed by geese and sediment accretion, and compared these with trends in goose abundance and vegetation cover, using aerial photographs (1979–2008), elevation maps (1931–2010), time series of goose counts (1987–2011) and vegetation maps (1979–2010).

## Methods

### Study site

**Fig 1:**
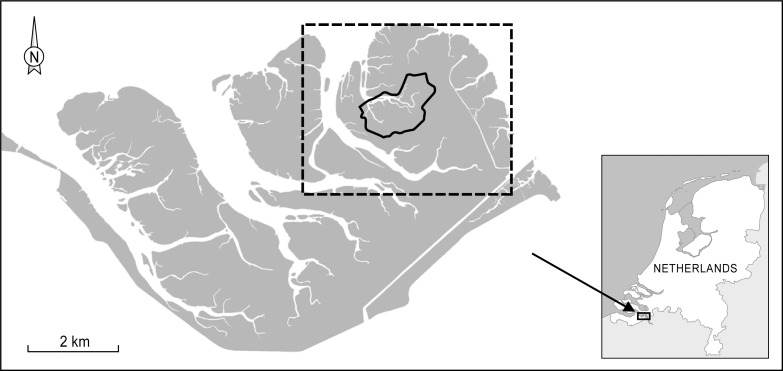
Location of the study area. The study area is a large brackish marsh, 28 km^2^ in size, located in the south of the Netherlands. The Greylag goose counts and vegetation cover were estimated for the entire marsh area. The solid line indicates the area where we measured the sediment accretion rate (approximately 2 km^2^), and the dashed line indicates the area where we studied regeneration of the depressions (approximately 7 km^2^).

The study area, Saeftinghe, is located in the Western Scheldt estuary in the Netherlands (Fig 1, 51°21’N, 4°11’E). It has a semidiurnal tidal regime with a mean tidal range of 4.9 m, and salinity varies between 5–18 PSU [49,50]. Permission to conduct this study was issued by *Het Zeeuwse Landschap*, which is the authority responsible for the protection of this national park. This study did not involve any endangered or protected species. Saeftinghe is considered as one of the largest brackish marshes in Western Europe, approximately 28 km^2^ in size, and is an important feeding habitat for large populations of wintering Greylag geese, *Anser anser* [33,40]. While a small part of the marsh is grazed by cattle, the largest part is now abandoned but had been extensively grazed by sheep until 1993. Outside the cattle-grazed marsh, the most important vegetation types are dominated by *Phragmites australis*, *Elytrigia atherica* or *Bolboschoenus maritimus*. *P*. *australis* is mostly limited to the eastern part of Saeftinghe near the seawall, *E*. *atherica* mainly dominates the higher elevated levees bordering creeks and *B*. *maritimus* is mainly limited to the depressions between levees. This marsh has only become an important staging and wintering site for Greylag geese since the 1980s. Greylag geese prefer to feed on the below-ground storage organs of *B*. *maritimus* for which they grub into the marsh soil [38,51]. A study by Castelijns et al. [40] showed that during the winters between 1994 and 1997, the main food sources of Greylag geese in Saeftinghe consisted of the following: 49% tubers of *B*. *maritimus*, 33% above-ground plant parts of other marsh plant species, 10% agricultural plants (growing on arable fields adjacent to the marsh) and 8% seeds of *E*. *atherica*. They concluded this based on microscopic evidence found in fresh droppings that had been collected in the field together with field observations [40].

### Estimating the population dynamics of Greylag geese

Between 1987 and 2011, numbers of Greylag geese in Saeftinghe were estimated at monthly intervals from July until March of the following year. The majority of the geese arrive in October and almost all leave again by the end of February, except for a small breeding population that remains year round. Goose numbers were estimated for the entire marsh area (28 km^2^) (data provided by *Natuurberschermingsvereniging De Steltkluut*). Goose counts were performed by several groups of people walking pre-determined routes, thereby covering the entire marsh area. By walking these routes simultaneously, they prevented that the same geese get counted multiple times. To include estimates of the population before 1987 in our analyses, we used population sizes estimated in the literature, which were estimated in Saeftinghe at monthly intervals as well [33]. In addition, the numbers of Greylag geese were counted between 1980 and 2011, throughout the Netherlands, including nature reserves and farmland (that were known for being bird hotspots). These bird counts were performed between September and April the next year, in one pre-specified weekend and replicated each month (data provided by Sovon, Dutch Centre for Field Ornithology).

### Top-down control by geese grubbing at the local scale

**Fig 2:**
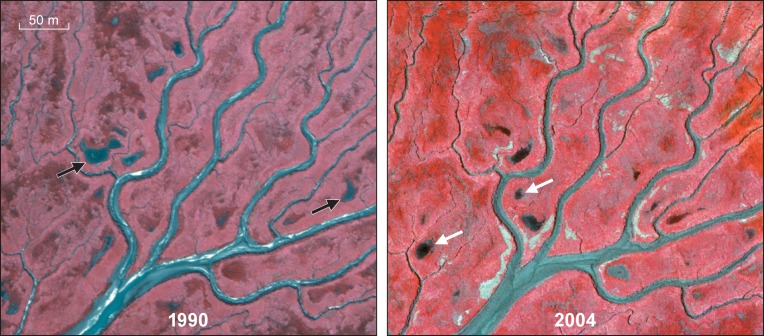
Occurrence of depressions in 1990 and 2004. Two representative aerial pictures comparing presence and absence of depressions in 1990 with 2004. The white arrows indicate depressions present in 1990 that had disappeared by 2004, and the yellow arrows point to depressions present in 2004 that were not yet present in 1990.

To determine the strength of top-down control by Greylag geese on the local vegetation, we used bare patches within the vegetation created through grubbing by Greylag geese and subsequent revegetation of these bare patches. Using ArcGIS, we analysed false-colour aerial photographs from 1979, 1990, 1998, 2004 and 2008. We identified all bare patches in the eastern region of Saeftinghe, an area approximately 7 km^2^ in size, for which the most central point was 51°21’45N, 4°11’46E (Fig 1). For each bare patch, we determined years that they were present as well as absent. Once the bare patch was no longer visible on the aerial photograph, we assumed vegetation had re-established at these bare patches (Fig 2). In this way, we could determine the minimum number of years that it had taken for vegetation to re-establish in each bare patch, i.e., the minimum regeneration time. For example, when a bare patch present in the photo taken in 1990 was no longer present in the photo taken in 2004 (Fig 2), we assumed revegetation of this bare patch had taken at least six years when we measured the vegetation composition in 2010. As we did not have aerial photographs for every year, we established four classes of minimum regeneration time: 0, 2, 6 and 12 years.

After determining the coordinates from the photographs, we visited these regenerated bare patches in the field in July and August of 2010. We measured the vegetation composition in 2 m x 2 m plots using the decimal scale of Londo [52]. In order to assess the impact of Greylag geese on the vegetation composition, we compared the vegetation composition in regenerated patches with the vegetation composition in depressions unaffected by geese. Thus, we measured vegetation composition in 13 control plots located in vegetation dominated by *B*. *maritimus*, where no visible signs of goose grubbing were present. As geese prefer to feed on the below-ground tubers of *B*. *maritimus*, we considered the *B*. *maritimus*-dominated vegetation type as the original vegetation type before geese activity resulted in the bare patches. As only three bare patches were recorded in the aerial photograph of 1979 that were no longer present in the aerial photograph of 1990 and therefore we excluded the minimum regeneration time of 20 years from further analyses. Additionally, we removed in total seven bare patches that got overgrown by expanding reed beds.

### Bottom-up control by sediment accretion at the landscape scale

To determine the strength of bottom-up control on the marsh vegetation exerted by vertical sediment accretion, and the subsequent increase of marsh elevation relative to sea level as well as its effects on the succession of *B*. *maritimus* vegetation towards other vegetation types, we measured both changes in vegetation type as well as long-term changes in surface elevation. To determine the cover of different vegetation types in Saeftinghe, we used vegetation maps generated by *Rijkswaterstaat* that had been produced using a widely practiced and validated method [53]. First, aerial photographs from 1979, 1998, 2004 and 2010 were analysed. Based on the false colour ranges, different putative vegetation types were identified. This was followed by multiple vegetation composition measurements performed in the field for each of the identified putative vegetation types. Ultimately, specific vegetation types were linked to colour ranges in the aerial photographs, and used to generate vegetation maps that overlapped the entire marsh.

Generally, the pioneer marsh harbours a combination of *Salicornia europaea* and *Spartina anglica*, which is replaced by species such as *Puccinellia maritima*, *Aster tripolium* and *Glaux maritima* [41,54]. Ultimately, the lower marsh becomes dominated by a cover of *B*. *maritimus* and the higher marsh by *E*. *atherica* and *P*. *australis*. In this study, we focused on the total cover of the three climax vegetation types, dominated by either *B*. *maritimus*, *E*. *atherica* or *P*. *australis*. When one of these three species was either dominant (at least 50% cover) or indicated as co-dominant in a vegetation type, we included that specific vegetation type in the analysis. All other vegetation types were excluded from the analysis.

The distribution of marsh plant species is determined for a large part by the local marsh surface elevation and the marsh surface increases in elevation as marshes become older. Therefore, an area of approximately 2 km^2^ was used to estimate long-term changes in marsh surface elevation (51°21’48N, 4°11’15E). Data were available for the years 1931, 1951, 1963, 1992, 2004 and 2010. Data were provided as Digital Terrain Models (DTMs) with a resolution of 20 m x 20 m for the years 1931, 1951, 1963 and 1992. These were based on topographic and bathymetric surveys performed by the Dutch and Belgian waterway management authorities [49,55]. Topographic surveys resulted in elevation data with a resolution of 1 measurement point per 7500 m^-2^. The elevations were mapped to the precision of 0.1 m relative to the Dutch Ordnance Level (NAP), which is close to mean sea level at the Dutch coast. This resulted in a maximum vertical error of ± 0.05 m. For the more recent time steps 2004 and 2010, DTMs with a resolution of 2 m x 2 m were available based on LIDAR data. These LIDAR surveys were carried out during low tide with a resolution ranging from 1 point per 16 m^-2^ to several points per m^-2^ and a vertical accuracy of ±0.2 m. The channel networks for 1931 and 2010 were merged and used as a mask to exclude grid cells located within the tidal channel network. The changes in the creek edges between the tidal channel networks of 1931 and 2010 were fairly limited because of the slow migration rate of the channels. Thus, we used this mask for all the time steps, as the creek edges in the intervening time steps between 1931 and 2010 would also be located within this mask. Besides the mean platform elevation of the selected site, the standard deviation was also calculated to represent the spatial variation in the marsh platform elevation. Historical data on mean high water level (MHWL) and mean high water level at spring tide (MHWLS) were derived from the nearby tidal gauge station at Bath, the Netherlands. The marsh surface elevation and the levels of MHWL and MHWLS are expressed in metres above NAP.

### Data analyses

To link the number of bare patches to the number of geese, we calculated the number of bare patches as well as the number of geese per unit marsh surface area (km ^2^). We first determined the maximum number of geese for the wintering season of each year, i.e., starting from October until March the following year. As geese grub during the winter and the number of bare patches is based on aerial photographs taken in summer, we averaged the maximum number of geese counted in the two years prior to the year when the number of bare patches was identified. To test whether there was a positive relationship between the number of patches and the number of goose we used a linear regression, using number of bare patches calculated per km^2^ as the response variable and the number of geese per km^2^ as the predictor variable. Many of the datasets used in this study were limited to singular measurements for each year (i.e. the cover of dominant vegetation types, the number of bare patches and the goose counts) and this limited the statistical analyses that could be performed. Cover of each plant species in the regenerating bare patches was compared with cover of that same species in the control plot. As many of these comparisons contained unequal variances and unequal sample sizes we used two-tailed Welch t-tests. A p-value < 0.05 was considered to indicate significant differences. All statistical analyses were performed in R version 3.3.1.[56]

## Results

### Estimating the population dynamics of Greylag geese

**Fig 3:**
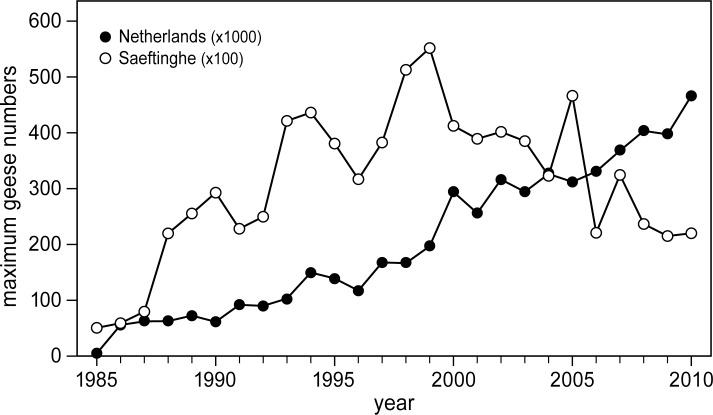
Number of wintering Greylag geese. Maximum number of wintering Greylag geese, estimated annually for the whole of the Netherlands (± 41.500 km^2^) and only Saeftinghe (± 28 km^2^). Maximum numbers were estimated at monthly intervals between fall and the spring of the following year. Goose numbers should be multiplied x1000 for the Netherlands and x100 for Saeftinghe (data from Sovon, the Dutch Centre for Field Ornithology, and *Natuurbeschermingsvereniging De Steltkluut*). Data between 1985 and 1987 for Saeftinghe were obtained from literature [33].

After hunting was abandoned in 1990, the population size of wintering geese in Saeftinghe increased substantially, from approximately 5,000 in 1985 to over 50,000 geese in 1998–1999 (Fig 3). Thereafter, the number of geese decreased again. Meanwhile, the number of Greylag geese in the Netherlands has still been increasing (Fig 3).

### Top-down control by geese grubbing at the local scale

**Fig 4:**
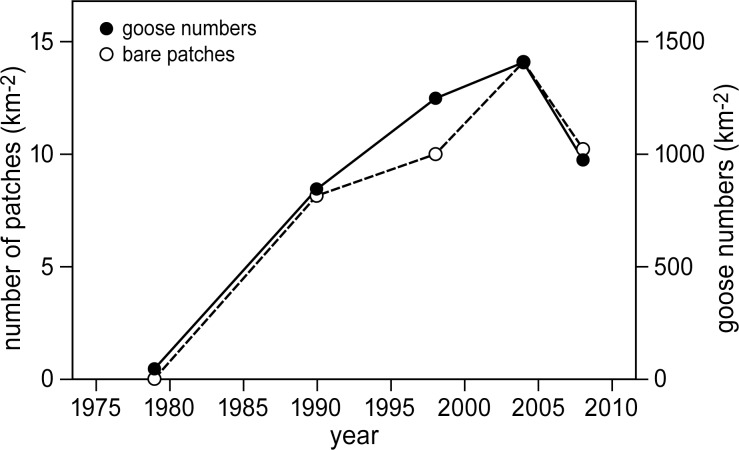
Changes in numbers of bare patches and goose numbers. Changes in the number of bare patches and the number of Greylag geese over time. Numbers of geese were determined by the maximum number of geese counted for the entire marsh area (28 km^2^), and these were averaged over the two years prior to the year that the number of bare patches was determined. The number of bare patches was determined only for the eastern part of the marsh (approximately 7 km^2^).

The total number of bare patches and the population size of the Greylag goose showed similar trends (Fig 4). That is, both increased until 2004 and similarly decreased after 2004, showing a strong positive linear relationship: number of bare patches per km^2^ = 0.008 * number of geese per km^2^ + 0.01 (adj. R^2^ = 0.88, F_1,3_ = 30.3, p = 0.01). On a marsh area of approximately 7 km^2^, we identified 9, 7 and 29 bare patches that were re-vegetated by 1998, 2004, and 2008, respectively. 71 bare patches were still unvegetated in 2008 (Table 1). Most of them were between 4 and 6 m in diameter, with some exceptions ranging up to 25 m in diameter.

**Table 1:** Cover of all plant species after re-establishment in bare soil. Vegetation cover (%) estimated per 2 m x 2 m in bare patches after 0, 2, 6 and 12 years of vegetation re-establishment (mean± SE). Control plots were estimated in *Bolboschoenus maritimus*-dominated vegetation.

Years after vegetation re-established					Control plot
	0	2	6	12	
Sample size (n)	71	29	7	9	13
Bare soil	74 ± 3	41 ± 5	21 ± 8	19 ± 7	12 ± 4
Litter	1 ± 0	3 ± 2	3 ± 3	18 ± 6	12 ± 4
*Agrostis stolonifera*	1 ± 1	5 ± 2	18 ± 10	6 ± 3	10 ± 5
*Aster tripolium*	8 ± 2	28 ± 5	13 ± 8	8 ± 5	0
*Atriplex prostrata*	1 ± 1	5 ± 2	3 ± 2	4 ± 2	9 ± 3
*Bolboschoenus maritimus*	2 ± 1	2 ± 1	17 ± 8	38 ± 4	46 ± 4
*Elytrigia atherica*	0	4 ± 3	13 ± 8	15 ± 6	11 ± 4
*Glaux maritima*	2 ± 1	0	6 ± 4	0	0
*Juncus gerardii*	0	0	0	0	1 ± 1
*Puccinellia maritima*	6 ± 2	11 ± 3	5 ± 3	2 ± 2	0
*Salicornia europaea*	6 ± 2	3 ± 2	7 ± 6	0	0
*Spartina anglica*	0	0	0	0	3 ± 3
Total cover	25 ± 3	57 ± 5	79 ± 8	64 ± 8	76 ± 4

**Fig 5:**
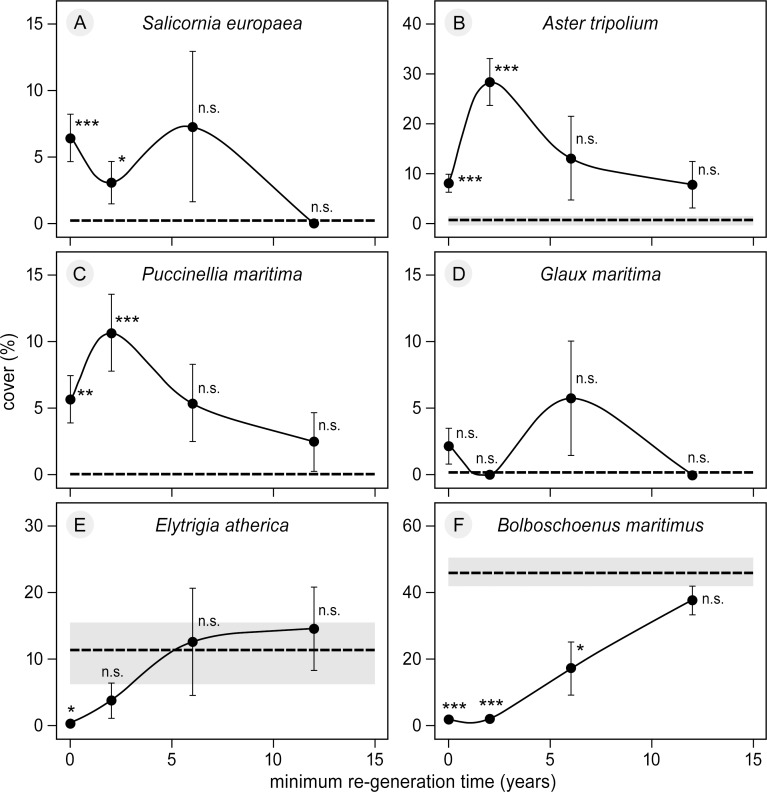
Cover of six plant species that established in bare patches. Cover of six plant species that established in the bare patches (mean± SE), shown in relation to the minimum number of years of regeneration, i.e., re-vegetation of the initially bare patches. Please note the different y-axes for different species. Data are shown for the different time classes of regeneration, i.e. after 0, 2, 6 and 12 years. Average cover of each species in the undisturbed control plots in the *Bolboschoenus maritimus*-dominated vegetation is indicated with a black broken line surrounded by the standard error (grey area). For each point the level of significance between bare patch and control plots are indicated (* = p < 0.05, ** = p < 0.001, *** = p < 0.001, n.s. = not significant).

Several early-successional species were found in the bare patches that were not present in the control plots (Table 1, Fig 5). For example, *Salicornia europaea* was observed as one of the first species to re-establish in the bare patches (Fig 5A). We found significantly higher cover of *S*. *europaea*, *Aster tripolium* and *Puccinellia maritima* in the bare patches compared to the control plots (Fig 5B and 5C). *E*. *atherica* and *B*. *maritimus* gradually re-colonized the bare patches and, eventually, their cover returned to similar levels as those in the control plots (Fig 5E and 5F). *E*. *atherica* reached a similar cover of approximately 11% after six years of regeneration, while *B*. *maritimus* reached a similar cover of approximately 50% after twelve years of regeneration. None of the plant species recorded in the re-vegetated plots showed a significant difference after twelve years (Fig 5).

### Bottom-up control by sediment accretion at the landscape scale

**Fig 6:**
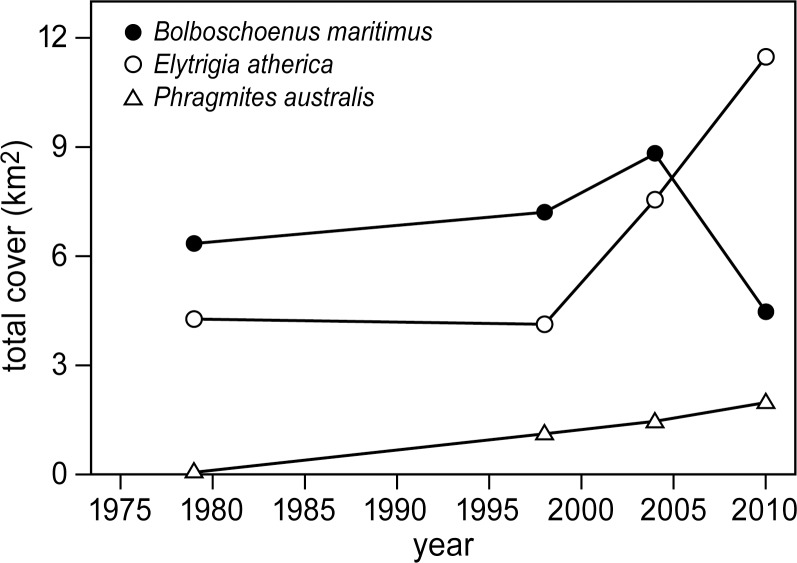
Changes in cover of dominant vegetation types. Changes in total cover over time of the three dominant vegetation types (*Bolboschoenus maritimus*, *Elytrigia atherica* and *Phragmites australis*), estimated for the entire marsh area. The cover of each plant species is given in km^2^ per total marsh area (28 km^2^).

Total cover of the three late successional vegetation types all clearly showed an increase from 1979 to 2004 (Fig 6). However, between 2004 and 2010, both *P*. *australis* and *E*. *atherica* vegetation types continued to increase, whereas the *B*. *maritimus* vegetation type decreased in cover from 8.8 km^2^ to 4.6 km^2^. Thus, similar changes over time were found in the *B*. *maritimus* vegetation cover (Fig 6), the number of geese and the number of bare patches (Fig 4). They all increased to a maximum in 2004 and decreased thereafter.

**Fig 7:**
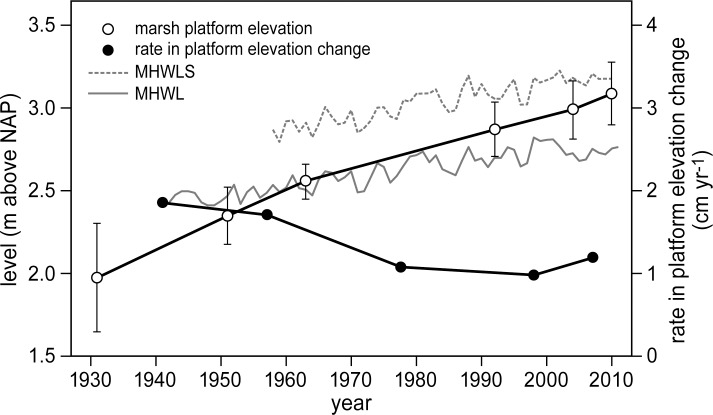
Changes in platform elevation and rate of elevation change. Marsh platform elevation change (m) and rate of elevation change (cm yr^-1^) in relation to Mean High Water Level (MHWL) and Mean High Water Level during spring tides (MHWLS). All levels are shown relative to the Dutch Ordnance Level (NAP). MHWLS data has only been available since the late 1950s.

The surface elevation of the marsh platform strongly increased over time at a rate that was faster than the increase in mean high water level (MHWL) (Fig 7). The rate of change in marsh platform elevation has decreased from 2 cm yr^-1^ to 1 cm yr^-1^ in the past few decades, but this is still higher than the average 0.4 cm yr^-1^ increase found in MHWL between 1930 and 2010. Thus, the marsh evolved from a low tidal marsh (mean platform elevation 0.47 m below MHWL) to a more highly elevated tidal marsh (mean platform 0.31 m above MHWL) from 1931 to 2010. Through natural succession, the vegetation composition also changed accordingly, i.e., increased cover of the *E*. *atherica* and *P*. *australis* vegetation types, whereas cover of the *B*. *maritimus* vegetation type decreased.

## Discussion

Our results partially support our first hypothesis in that top-down control by geese grubbing on vegetation resulted in local degradation of the marsh surface and a reduction in the cover of *B*. *maritimus*. However, these effects were limited in time as the bare patches were able to revert back to the initial *B*. *maritimus* vegetation type within 12 years. This is in contrast to previous studies, where grubbing by Snow geese *Anser caerulescens* caused large-scale marsh degradation that continued even after the geese had disappeared [36,37]. Our results, however, fully support our second hypothesis in that bottom-up control exerted at the landscape-scale by sediment accretion on vegetation development resulted in a higher elevation and reduction in the overall *B*. *maritimus* cover over the course of 30 years of vegetation succession. Our results show that top-down and bottom-up controls can co-occur but at different spatial as well as temporal scales. Overall, we conclude that, in the long term, vegetation composition is primarily controlled by bottom-up controls driven by sediment accretion, rather than through top-down control by geese.

### Top-down control by geese grubbing at the local scale

On a local patch scale of a few square metres, grubbing by Greylag geese was shown to exert top-down control on the vegetation by creating bare patches. This is in line with previous studies showing that grubbing by geese can have strong negative effects on coastal habitats [35,38,57,58]. However, in contrast to these studies, we found re-vegetation of the patches back to a similar vegetation type dominated by *B*. *maritimus* (Fig 5), whereas some studies concluded that regeneration might not even be possible [37,39,57]. The effect of grubbing depends on spatial scale. Large-scale removal of vegetation can change important ecosystem processes which may retard or even prevent re-generation [36,37,39]. Whereas on a small scale, presence of goose grubbing in our study only resulted in a local, temporary setback of vegetation succession, which positively affected plant diversity. This result is consistent with several previous studies showing that grazers, such as livestock, can bring back or maintain earlier successional plant species in older marsh systems [59,60].

The global increase in migrating goose populations has resulted in expanding grazing pressure on many tidal marshes [29,31,32,36]. As mentioned previously, the impact of geese grubbing differed between marshes, ranging from large-scale, long-term vegetation disturbance [36,57] to small-scale, short-term disturbance (e.g. this study). The outcome of these two possibilities could depend on local abiotic stress as well as on the heterogeneity present within the ecosystem. First, abiotic conditions that are known to exert stress on plant growth in tidal marshes, such as high salinity [58,61], low marsh elevation relative to mean sea-level [18] and low tidal range [47,62], can all limit successful (re-)establishment of marsh plant species. Our study site is a relatively high productive, brackish marsh (5–18 PSU). Additionally, it has a large tidal range (4.9 m on average) [49] and an average surface elevation that is located high in the tidal frame, i.e., between mean high water level and mean spring high water level (see also Fig 7). Re-colonization by early successional plant species in bare patches will be less limited in our study site compared with less productive, more saline marshes with low tidal ranges and low elevation relative to mean sea level. Second, Saeftinghe is a very heterogeneous marsh. Its marsh surface is drained by dense networks of creeks, which are bordered by higher elevated levees dominated by *E*. *atherica*, alternating with small depressions (20–30 cm lower than the levees) covered by *B*. *maritimus* [50,55]. When geese grub for below-ground tubers, they generally continue until their preferred food choice is depleted [36,38]. According to other studies, this foraging behaviour has resulted in degradation of large areas of marsh [36–39]. Because of the patchy and heterogeneous structure of Saeftinghe, *B*. *maritimus* is present in smaller patches within depressions surrounded by *E*. *atherica*, which are less preferred by the geese. This forces the geese to continuously move on to new unexplored patches of *B*. *maritimus* and thereby limits their detrimental impact on the marsh vegetation. Thus, the impact of goose grubbing could be marsh-specific and may depend on many different environmental conditions, such as the local abiotic conditions and level of heterogeneity.

The number of bare patches was strongly linked to the number of Greylag geese (Fig 4), and we assumed in this study that all patches were the result of goose grubbing. However, we used a time-series of aerial photographs to identify the patches and most were re-vegetated by the time we visited them in the field. The geese had already abandoned them at this point so that we could not directly link the formation of these patches to grubbing by Greylag geese. Other disturbances can cause bare patches to form in the marsh vegetation, such as marsh submergence by relative sea-level rise [63], erosion [64] and presence of dead plant material deposited on the marsh surface during storms [65,66]. However, when we visited our field site, we observed many locations in different stages of degradation, including total lack of vegetation. Here, we found clear evidence for the presence of Greylag geese grubbing for the tubers of *B*. *maritimus*. We observed multiple holes of ~5 cm deep and ~3cm in diameter that could have been caused by geese inserting their bills into the marsh soil, and goose droppings surrounding these newly formed bare patches. Furthermore, Leemans and Verspaandock [67] did not mention the presence of bare patches when they described the vegetation of our study site in 1971, and we identified only three bare patches in the aerial photograph of 1979 when few Greylag geese were present (Fig 4). We cannot exclude the possibility that some of the bare patches were the result of some other type of disturbance. However, based on our own field observations and evidence provided by other studies documenting that grubbing by waterfowl can create these types of patches in the marsh surface, we find that it is a reasonable assumption that most bare patches in our field site were the result of grubbing by Greylag geese [35].

### Bottom-up control by sediment accretion at the landscape scale

The marsh surface was shown to increase rapidly due to high accretion rates that outpaced local sea-level rise (Fig 7). *B*. *maritimus* generally dominates the lower elevated depressions in between creek banks with waterlogged conditions [54,68] and is the preferred food choice for Greylag geese [40,69]. In contrast, *E*. *atherica* has been shown to be limited to higher elevated creek banks without waterlogged conditions [18,45]. A slow increase in surface elevation generally results in vegetation succession to occur and this would benefit all climax species, including *B*. *maritimus*. However, a continued increase in surface elevation that coincides with a reduction in waterlogged conditions would ultimately enable the highly competitive grass *E*. *atherica* to expand from the higher elevated levees to the lower elevated depressions [70], thereby reducing cover of *B*. *maritimus*. This would also explain the slow increase followed by the sharp drop in *B*. *maritimus* cover that we found. In the 1990s, accretion rates between 2 and 4 mm yr^-1^ were measured in the back-barrier salt marsh of Schiermonnikoog, the Netherlands [71]. Here, *E*. *atherica* also spread at the cost of low-statured species such as *Puccinellia maritima* and *Festuca rubra* [45]. The reduction of these edible plant species initially resulted in a decline of winter-staging Brent geese *Brant bernicla* [17], followed by a decrease in European brown hares *Lepus europaeus* [72]. Similarly to these studies, we observed a reduction in Greylag goose numbers that co-occurred with an increase in *E*. *atherica* at our study site.

### Population dynamics of Greylag geese

In the last decade, both the number of Greylag geese and the cover of *B*. *maritimus* have decreased in this marsh (Figs 3, 4 and 6). Local removal of *B*. *maritimus* vegetation by Greylag geese, combined with continued vegetation succession to other climax vegetation types due to sediment accretion, will negatively affect their food supply, namely the cover of *B*. *maritimus*. Furthermore, not all *B*. *maritimus* vegetation present in the ecosystem can be reached by Greylag geese. First, they need a local disturbance in the otherwise tall and dense vegetation so that they can land in the disturbed area and use such spots to access the *B*. *maritimus* food source [38]. In our study site, *E*. *atherica* surrounding the *B*. *maritimus* patches will allow the geese to land and access the patches from the sides. Second, Greylag geese can only forage on small, newly developed tubers [69,73]. During foraging they will remove the entire plant with the attached tubers from the soil and eat the newly developed tubers, while discarding the larger old tubers causing bare patches to form. Last, grubbing by wintering Greylag geese might be limited to lower elevated depressions with water-logged conditions. Such conditions soften the soil and allow for easier access for geese to grub into the soil [38,51]. This also explains why the bare patches in our study site were generally situated near creeks in the depressions where geese can enter the waterlogged soil and reach the belowground tubers of *B*. *maritimus*. A landscape-scale increase in the marsh platform elevation, as observed in Saeftinghe, generally decreases tidal inundation and soil water logging, which in turn increases soil compaction [74]. This will further reduce the number of *B*. *maritimus* patches accessible for geese.

In the previous section, we linked the reduced number of wintering Greylag geese to the decreasing food supply. However, there are alternative explanations for this trend. First, Red foxes *Vulpes vulpes* have started to disturb nests of breeding Greylag geese in spring [75]. Greylag geese began breeding in the study area in the early 1990s. The number of observed nests amounted to 72 in 1997, 312 in 2004 and 335 in 2012. Around 60% of the nests failed in 2014 as a result of predation by foxes [75]. Nevertheless, top-down regulation of wintering goose numbers by foxes seems unlikely, as their numbers had already started to decline from 1999 onward (Fig 3), and they are much less vulnerable to predation than breeding geese. A second explanation could be a cascade effect of reduced breeding success at their breeding habitat. However, the numbers of Greylag geese throughout the Netherlands has continued to increase (Fig 3). Greylag geese wintering in Germany, Spain and the Netherlands breed together in northern Europe [30,76,77]. A reduction in goose numbers at our study site resulting from reduced breeding success or reduced survival along their migrating route would have resulted in reduced populations throughout the Netherlands. It would also have affected populations in other European countries, such as Germany and Spain [76,78]. As this is not the case [77,79], it is much more likely that our study site has become less suitable for sustaining a large number of Greylag geese because of sediment accretion followed by changes in vegetation composition and subsequent reduction in food supply.

### Top-down vs. bottom-up control and the importance of scale

The bottom-up control by sediment accretion on the vegetation dynamics was more important (i.e. acted on larger spatial and temporal scales) than the top-down control exerted by goose grubbing. We had actually expected to find a much larger effect by goose grubbing. The local effects by geese grubbing on the marsh vegetation may even disappear at a faster rate because of increasing marsh elevation. Sediment accretion could expedite vegetation succession and this may overrule the successional regression by geese, thereby reducing even the local impact by Greylag geese. Thus, we showed that both top-down and bottom-up control on the vegetation occurred simultaneously but at very different spatial and temporal scales. Based on the observed increase in marsh surface elevation and the natural succession towards *E*. *atherica*, we conclude that the vegetation dynamics were primarily controlled by bottom-up control exerted by sediment accretion at the landscape-scale.

This study underlines the complexity of determining the factors that control vegetation dynamics in an ecosystem. Studies focusing on the interactive effects of top-down control by the grazers and bottom-up control by resources on the vegetation generally do not take spatial or temporal scale into account [2,24]. In this study, we showed the importance of including larger spatial as well as temporal scales to study important ecosystem dynamics. In most studies this would be difficult due to constraints in space and time. The long-term datasets used in this study gave us the unique opportunity to study top-down versus bottom-up control of the vegetation dynamics within the same system and to include large spatial and temporal scales. However, the large time steps and limited number of aerial photographs reduced our ability to test the mechanisms of top-down and bottom-up control more rigorously. Most importantly, we could not directly link sediment accretion to the cover of the different vegetation types. However, it has been shown that elevation and inundation are the most important factors controlling vegetation dynamics in tidal marshes [18,41], and these two factors are directly linked to sediment accretion. In this study we attribute the reduction in *B*. *maritimus* between 2004 and 2010 to sediment accretion. An alternative explanation for this could be the extremely high numbers of geese observed in 2005 (Fig 3), i.e. top-down control. The *B*. *maritimus* vegetation could still be recovering from this impact when it was estimated in 2010. However, this alternative explanation is unlikely as equal or higher numbers of Greylag geese were also present in the years 1993–1994 and 1998–1999. Most patches grubbed by the geese in these years should have recovered by 2010, which would counterbalance the newly grubbed bare patches in 2005. Furthermore, the total area covered by the patches is only a fraction of the total cover of *B*. *maritimus*. We do propose that the mechanisms underlying the correlations found in this study need further investigation. The next steps should be the use of manipulative field experiments, including long-term exclosure experiments to remove grubbing by Greylag geese. Re-generation time of the bare patches may also depend on initial patch size [80], and this should be investigated in more detail. The change in vegetation type as well as the local sedimentation rate should be measured in permanent plots in lower elevated depressions to test that a change in surface elevation due to sedimentation is indeed controlling the vegetation bottom-up.
